# In-depth quantitative proteomic characterization of organotypic hippocampal slice culture reveals sex-specific differences in biochemical pathways

**DOI:** 10.1038/s41598-021-82016-7

**Published:** 2021-01-28

**Authors:** Simone Nardin Weis, Jaques Miranda F. Souza, Juliana Bender Hoppe, Marina Firmino, Manfred Auer, Nassim N. Ataii, Leonardo Assis da Silva, Mariana Maier Gaelzer, Caroline Peres Klein, Alan R. Mól, Consuelo M. R. de Lima, Diogo Onofre Souza, Christianne G. Salbego, Carlos André O. Ricart, Wagner Fontes, Marcelo Valle de Sousa

**Affiliations:** 1 Laboratory of Protein Chemistry and Biochemistry, Department of Cell Biology, Institute of Biology, University of Brasília, Brasília, DF 70910-900 Brazil; 2 Department of Biochemistry, Federal University of Rio Grande do Sul, Porto Alegre, 90035-003 Brazil; 3 Molecular Biophysics and Integrated Bioimaging Division, Lawrence Berkeley National Laboratory, 1 Cyclotron Road, MS Donner, Berkeley, CA 94720 USA; 4 Laboratory of Electron Microscopy, Department of Cell Biology, Institute of Biological Sciences, University of Brasília, Brasília, DF 70910-900 Brazil; 5 University of Miami, Miller School of Medicine, Miami, FL USA

**Keywords:** Neurochemistry, Proteomics

## Abstract

Sex differences in the brain of mammals range from neuroarchitecture through cognition to cellular metabolism. The hippocampus, a structure mostly associated with learning and memory, presents high vulnerability to neurodegeneration and aging. Therefore, we explored basal sex-related differences in the proteome of organotypic hippocampal slice culture, a major in vitro model for studying the cellular and molecular mechanisms related to neurodegenerative disorders. Results suggest a greater prevalence of astrocytic metabolism in females and significant neuronal metabolism in males. The preference for glucose use in glycolysis, pentose phosphate pathway and glycogen metabolism in females and high abundance of mitochondrial respiration subunits in males support this idea. An overall upregulation of lipid metabolism was observed in females. Upregulation of proteins responsible for neuronal glutamate and GABA synthesis, along with synaptic associated proteins, were observed in males. In general, the significant spectrum of pathways known to predominate in neurons or astrocytes, together with the well-known neuronal and glial markers observed, revealed sex-specific metabolic differences in the hippocampus. TEM qualitative analysis might indicate a greater presence of mitochondria at CA1 synapses in females. These findings are crucial to a better understanding of how sex chromosomes can influence the physiology of cultured hippocampal slices and allow us to gain insights into distinct responses of males and females on neurological diseases that present a sex-biased incidence.

## Introduction

In mammals, *sex* refers to the biological variable determined by XX chromosomal complement, while the construction of *gender* is influenced by a particular cultural and social context that defines feminine and masculine^1,2^. Different responses to therapies and susceptibility to a specific disease are considered sex differences^2^. Women show greater susceptibility to autoimmune diseases, including multiple sclerosis, rheumatoid arthritis and systemic lupus erythematosus, and sex hormones and/or sex-linked gene inheritance are responsible for this^3^. Women are more prone to develop age-related neurodegenerative diseases, such as Alzheimer’s disease and other dementias^4,5^, and they present a higher risk for major depression, post-traumatic stress disorder, and anxiety disorders^6^. In contrast, men are more likely to develop schizophrenia, autism, Parkinson’s disease^6^, and more susceptible to genetic disorders that cause intellectual disabilities, largely due to the prevalence of X-linked genes in the brain that mediate neural development and function^7^.

Magnetic resonance spectroscopy of the excised human epileptogenic hippocampus identifies high glutamate and lactate levels and low glutamine/glutamate ratio, an indication for poor glucose use that could lead to the worsening of energy metabolism^8^. In the left hippocampus from bipolar I disorder patients, decreased concentrations of N-acetylaspartate + N-acetyl-aspartyl-glutamate (markers of neuronal integrity) and creatine + phosphocreatine (marker of energy buffer capacity) were associated with microglial activation and mood symptoms^9^. Multiple metabolic pathways, such as glycolysis, TCA cycle, oxidative phosphorylation and phosphocreatine system were impaired in the hippocampus of APP/PS1 transgenic mice with Alzheimer’s disease. These changes were accompanied by altered fatty acid profile, suggesting a deregulation in the biosynthesis, turnover and acyl chain remodeling of phospholipids^10^. When mitochondrial function was evaluated in the hippocampus of neonatal rats submitted to hypoxia–ischemia, females presented greater mitochondrial activity overall than males and also proved to be more vulnerable to the injury^11^. This mitochondrial dysfunction was followed by activation of autophagy, which could represent a survival response^12^.

There is a large body of evidence linking molecular and cellular deregulation to poor physiological outcome in many neurological disorders. In this context, organotypic slices have been increasingly used as a major in vitro model for studying the cellular and molecular mechanisms related to neurodegenerative disorders that present a sex-biased incidence, such as Alzheimer’s and Parkinson’s diseases^13^. For this reason, it is critical to investigate the impact of sex chromosomes on the normal physiology of cultured slices, so that we can better understand how and why males and females are affected differently by such diseases.

The large-scale comparison of proteome profiles using liquid chromatography-tandem mass spectrometry (LC–MS/MS) from the hippocampus of females and males enables us to identify specific cellular processes implicated in sex differences that might reflect physiological features. Ultimately, it may allow the enhancement of accuracy and effectiveness in the clinical care of patients, personalizing healthcare, especially in those diseases with a sex component.

## Results

In order to identify possible specific proteins or metabolic pathways in the normal function of the hippocampus that may differ in females and males, an in-depth proteome quantitative comparison was carried out, along with transmission electron microscopy imaging and flow cytometry analysis.

### Label-free quantitative analysis of hippocampal slice culture

#### Proteins identified and quantified

A total of 4615 proteins in 3329 groups, with at least two unique peptides and a protein false discovery rate of less than 1%, were identified. Among them, 1286 protein entries from UniprotKB were significantly regulated, with approximately 63% of them more abundant in females and 37% in males. Pearson's correlation coefficient between biological replicates in males and females showed similar biological variability (each biological replicate means one animal) between the replicates within each condition across the clusters (Suppl. Figure 1).

**Figure 1:**
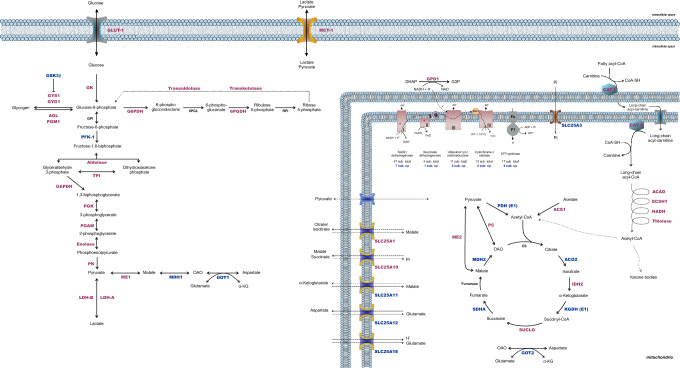
Differentially expressed proteins associated with energy metabolism pathways in rat hippocampus. Proteins in pink represent increased abundance in female hippocampus and proteins in blue represent increased abundance in male hippocampus (n = 5 biological replicates, each containing 3 slices). To simplify understanding, the number of upregulated subunits of mitochondrial respiratory chain complexes was indicated below the number of total subunits of each complex. For more details, see Supplementary Dataset 1. Statistical analyses were performed using Student's *t* test followed by multiple testing corrections using the Benjamini–Hochberg procedure for false discovery rate.

#### Identification of enriched pathways

KOBAS was applied to identify the most enriched pathways among the differentially abundant proteins, given a background distribution using the whole proteome identified in each group (male or female). Functional pathway annotation results indicated that the levels of metabolism of xenobiotics by cytochrome P450 (corrected *p* = 0.00256, 71%), fatty acid degradation (corrected *p* = 0.00081, 59%), and glutathione metabolism (corrected *p* = 0.00299, 57%) were the most significantly enriched terms in the female group. In the male group, the terms synaptic vesicle cycle (corrected *p* = 0.00017, 36%), and Alzheimer’s, Parkinson’s and Huntington’s diseases were enriched (corrected *p* = 4.65E−06—30%, corrected *p* = 0.00064—27% and corrected *p* = 0.00090—24%, respectively), among a long list of enriched terms (Suppl. Figure 2).

**Figure 2:**
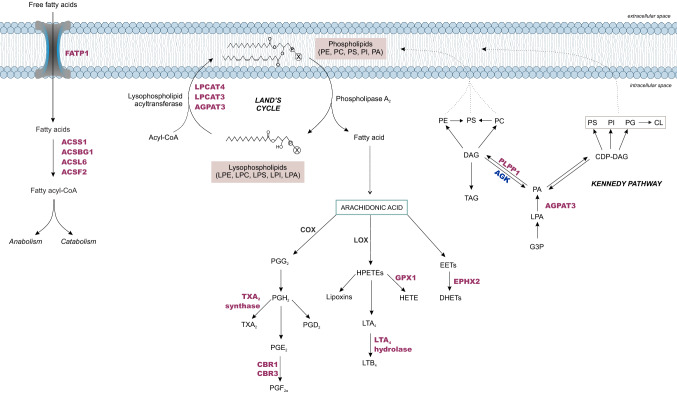
Biosynthetic pathways of glycerophospholipids in rat hippocampus. Glycerophospholipids are synthetized in the Kennedy pathway and subsequently remodeled in the Lands cycle, contributing to lipid diversity and the production of intracellular lipid mediators. The differentially expressed proteins revealed by proteomics in organotypic hippocampal slice culture are highlighted in pink (increased abundance in female) and blue (increased abundance in males). For more details, see Supplementary Dataset 1. Statistical analyses were performed by using Student's *t* test followed by multiple testing corrections using the Benjamini–Hochberg procedure for false discovery rate (n = 5 biological replicates, each containing 3 slices).

#### Overview of glucose metabolism

Carbohydrate oxidation from glucose to pyruvate reveals that eight proteins were upregulated in females. The glycolytic enzyme phosphofructokinase-1 (PFK-1) was the only one upregulated in males. Both lactate dehydrogenase A and B subunits (LDH-A and LDH-B), which preferentially convert pyruvate to lactate and the opposite, respectively, were also increased in females (Fig. 1, Suppl. Dataset 1). Isoform 1 of the glucose transporter (GLUT1), localized in endothelial cells and astrocytes, was almost twofold increased in females. GLUT-1 density is accompanied by abundance of high astrocytic plasma membrane monocarboxylate transporter 1 (MCT-1) in females. Several isoforms of a mitochondrial carrier family (SLC25 family of transporters) were identified.

Pyruvate dehydrogenase E1 component (PDH-E1) from the PDH complex, which catalyzes the overall conversion of pyruvate to acetyl-CoA and CO_2_, increased more than twofold in males. In the TCA cycle, isocitrate dehydrogenase (IDH2) and succinate-CoA ligase (SUCLG) were more abundant in females. On the other hand, aconitase (ACO2), α-ketoglutarate dehydrogenase (KGDH-E1), succinate dehydrogenase (SDHA) and malate dehydrogenase (MDH2) increased in males. The cytosolic isoform of malate dehydrogenase (MDH1) was also upregulated in males. The abundance of anaplerotic enzymes, both cytosolic and mitochondrial malic enzyme (ME1 and ME2, respectively) and pyruvate carboxylase (PC), an astrocyte-specific enzyme, were greater in females (Fig. 1).

Parallel to glycolysis, in the pentose phosphate pathway (PPP), a portion of the pentose carbon backbone can be rearranged to form glycolytic intermediates through the rate-limiting enzyme glucose-6-phosphate dehydrogenase (G6PDH), along with 6-phosphogluconate-dehydrogenase (6PGDH), transaldolase and transketolase, all predominantly increased in females. In astrocytes, G6P can be stored as glycogen. The content of glycogen synthase (GYS1) and glycogenin-1 (GYG1) involved in the synthesis of glycogen, as well as glycogen debranching enzyme (AGL) and phosphoglucomutase 1 (PGM1), necessary to break down glycogen to produce glucose, was higher in females (Fig. 1).

The ATP from oxidative phosphorylation is primarily utilized to support membrane potential, and essential cellular functions such as vesicle cycling, signal transduction, neurotransmitter synthesis, and axoplasmic transport^14,15^. Oxidative phosphorylation, the final stage of cellular respiration, is processed by the electron transport chain (ETC) and F_o_F_1_-ATP synthase. There was upregulation of some protein subunits in CI (7), CII (1), CIII (2), CIV (2) and ATP synthase (4) only in the male group (Fig. 1). In contrast, glycerol-3-phosphate dehydrogenase (GPD1), responsible for delivering cytosolic-reducing equivalents into ETC and regenerating cytosolic NAD^+^, increased in females. The upregulation of cytosolic creatine kinase B-type in females and mitochondrial creatine kinase U-type in males was also observed.

#### Lipid metabolism

Fatty acids (FAs) can be imported to the intracellular medium through transmembrane transporters, such as the long-chain fatty acid transport protein 1 (FATP1), which also possess acyl-CoA ligase activity. Whether used in anabolic or catabolic pathways, FAs must be activated by acyl-coenzyme A synthetases (ACSs) through thioesterification to CoA. The acyl-CoA synthetase short-chain family member 1 (ACSS1), acyl-CoA synthetase bubblegum family member 1 (ACSBG1), acyl-CoA synthetase long-chain family member 6 (ACSL6) and the acyl-CoA synthetase ACSF2 increased significantly in females (Fig. 2, Suppl. Dataset 1). In line with these results, oxidation of FA steps from carnitine-bound transportation to mitochondria through carnitine palmitoyltransferase 1 and 2 (CPT1 and CPT2, respectively) and the four steps of β-oxidation catalyzed by acyl-CoA dehydrogenase (ACAD), enoyl-CoA hydratase (ECSH1), hydroxyacyl-CoA dehydrogenase (HADH) and thiolase were all increased in females (Fig. 1).

Glycerophospholipids are essential components of cellular membranes in the brain. The acyl chains of these moieties are synthesized in the de novo pathway and subsequently remodeled by the action of phospholipases and lysophospholipid acyltransferases, allowing glycerophospholipid diversity and generation of lipid mediators^16^. Lysophospholipid acyltransferase 3 (LPCAT3), LPCAT4 and acylglycerol-3-phosphate acyltransferase 3 (AGPAT 3) were all upregulated in females. In the de novo synthesis, lipid phosphate phosphohydrolase 1 (PLPP1), which produces diacylglycerol from phosphatidic acid, was upregulated in females, while acylglycerol kinase (AGK), which catalyzes the reverse reaction, was upregulated in males (Fig. 2, Suppl. Dataset 1). Arachidonic acid (AA) is the initial substrate for lipid-derived mediators that function as signaling molecules. Females showed an upregulation of enzymes related to the generation of thromboxane A2 (TXA_2_ synthase), prostaglandin F2α (carbonyl reductase 1 and 3), leukotriene B4 (LTA_4_ hydrolase), hydroxyeicosatetraenoic acid (HETE—glutathione peroxidase 1) and dihydroxyeicosatrienoic acids (DHETs—bifunctional epoxide hydrolase 2) (Fig. 2).

#### Amino acid metabolism

With respect to differentially abundant proteins annotated in amino acid metabolism (Suppl. Dataset 4), it is important to highlight those related to metabolism of the neurotransmitters glutamate and γ-aminobutyric acid (GABA). These proteins are more fully described in the Discussion section  below (Suppl. Dataset 4, Fig. 7).

#### Redox homeostasis

**Figure 3:**
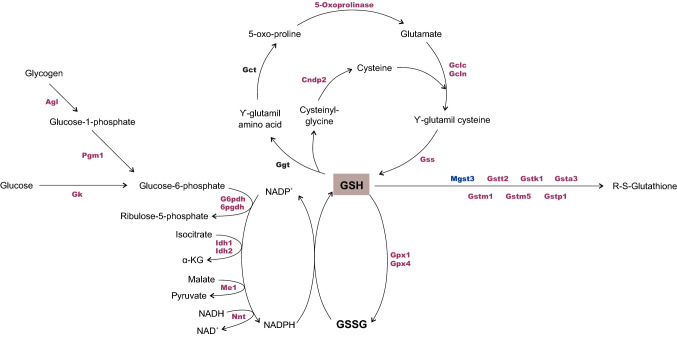
Glutathione metabolism. Proteomic analysis revealed prevalence of GSH synthesis and consumption in female hippocampus. The glutathione redox cycling catalyzed by GPx and GR is dependent on NADPH as a supplier of reducing equivalents, constantly regenerated mainly by pentose phosphate pathway. The differentially expressed proteins revealed by proteomics in organotypic hippocampal slice culture are highlighted in pink (increased abundance in female) and blue (increased abundance in males). For more details, see Supplementary Dataset 4. Statistical analyses were performed by using Student's *t* test followed by multiple testing corrections using the Benjamini–Hochberg procedure for false discovery rate (n = 5 biological replicates, each containing 3 slices).

A remarkable sex difference pattern was observed in the redox system, especially in glutathione (GSH) metabolism. Basically, most of the proteins involved in GSH synthesis, GSH conjugation to xenobiotics and in the NADPH-producing system present greater abundance in females (Fig. 3, Suppl. Dataset 4). Thioredoxin reductase 1 and peroxiredoxins (PRDX1, PRDX5 and PRDX6), related to cell defense against reactive oxygen/nitrogen species and redox homeostasis were upregulated in females (Suppl. Dataset 4). Proteins related to the mechanism of cell death are summarized in Suppl. Dataset 4.

#### Neurotransmitters and nervous system related proteins

**Figure 4:**
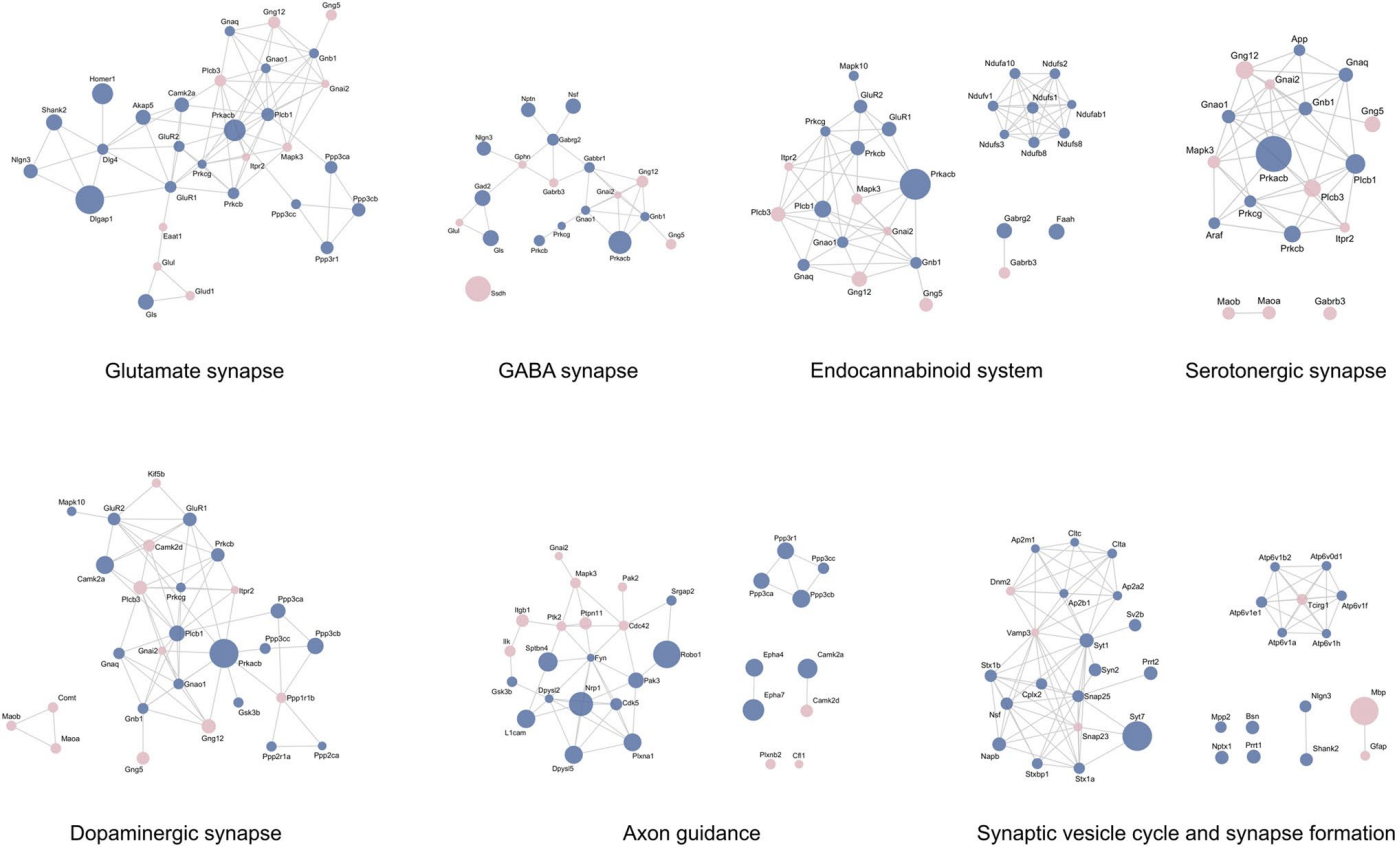
Molecular interaction network of proteins differently abundant between females and males underlying neurotransmission pathways and synapse function. Node colors indicate increased abundant proteins in female (pale pink) and male (blue) hippocampus, and node size indicates different levels of fold changes relative to each individual network. Protein–protein interaction analysis of significantly changed proteins in the hippocampus was obtained using STRING and then mapped by using Cytoscape 3.7.1. For more details, see Supplementary Dataset 3. Statistical analyses were performed by using Student's *t* test followed by multiple testing corrections using the Benjamini–Hochberg procedure for false discovery rate (n = 5 biological replicates, each containing 3 slices).

The KEGG pathway analysis related to the nervous system revealed that there is a slightly predominant distribution of proteins identified in the male compared to the female hippocampus (Suppl. Figure 3). The scaffold proteins in postsynaptic density (PSD) HOMER1 (3.26—FC), guanylate kinase-associated protein—GKAP (4.47—FC) and high-affinity calcium sensor synaptogamin-7, involved in exocytosis (7.37—FC) were highly upregulated in males. In females, the most upregulated protein was myelin basic protein—MBP (6.97—FC), essential for the formation of CNS myelin (Suppl. Figure 3). PSD proteins, mostly involved in glutamatergic synapses, were greatly abundant in males, including PSD-95 (DLG4), GKAP, HOMER1, CAMK2A, AKAP, SHANK2 and the ionotropic receptors GLUR1 and GLUR2 (Fig. 4). Proteins related to the axon guidance process were highly upregulated in males, such as ROBO1, the receptors tyrosine kinase Ephrin type-A receptor 4 and 7 (EPH4 and EPHA7, respectively), the semaphorin receptor plexin A1 (PLXNA1) and the Ca^2+^- and calmodulin-dependent serine/threonine protein phosphatase calcineurin (PPP3R1, PPP3CA, PPP3CB and PPP3CC subunits). The synaptic vesicle cycle and synapse formation pathways showed essential components associated with transport vesicle docking and fusion in presynaptic active zones, especially in males, such as bassoon (BSN), synaptogamin 1 (SYT1), syntaxins (STX1A, STX1B) and synaptosomal-associated protein 25 (SNAP25) (Fig. 4).

#### Proteins associated with neurodegenerative disorders

Sex differences are presented in most neurodegenerative disorders. In order to emphasize these differences, a few interesting and differentially abundant proteins noted with regard to the two most prevalent disorders, Alzheimer’s and Parkinson’s diseases (AD and PD, respectively), are represented in Supplementary Fig. 4.

#### Signal transduction

**Figure 5:**
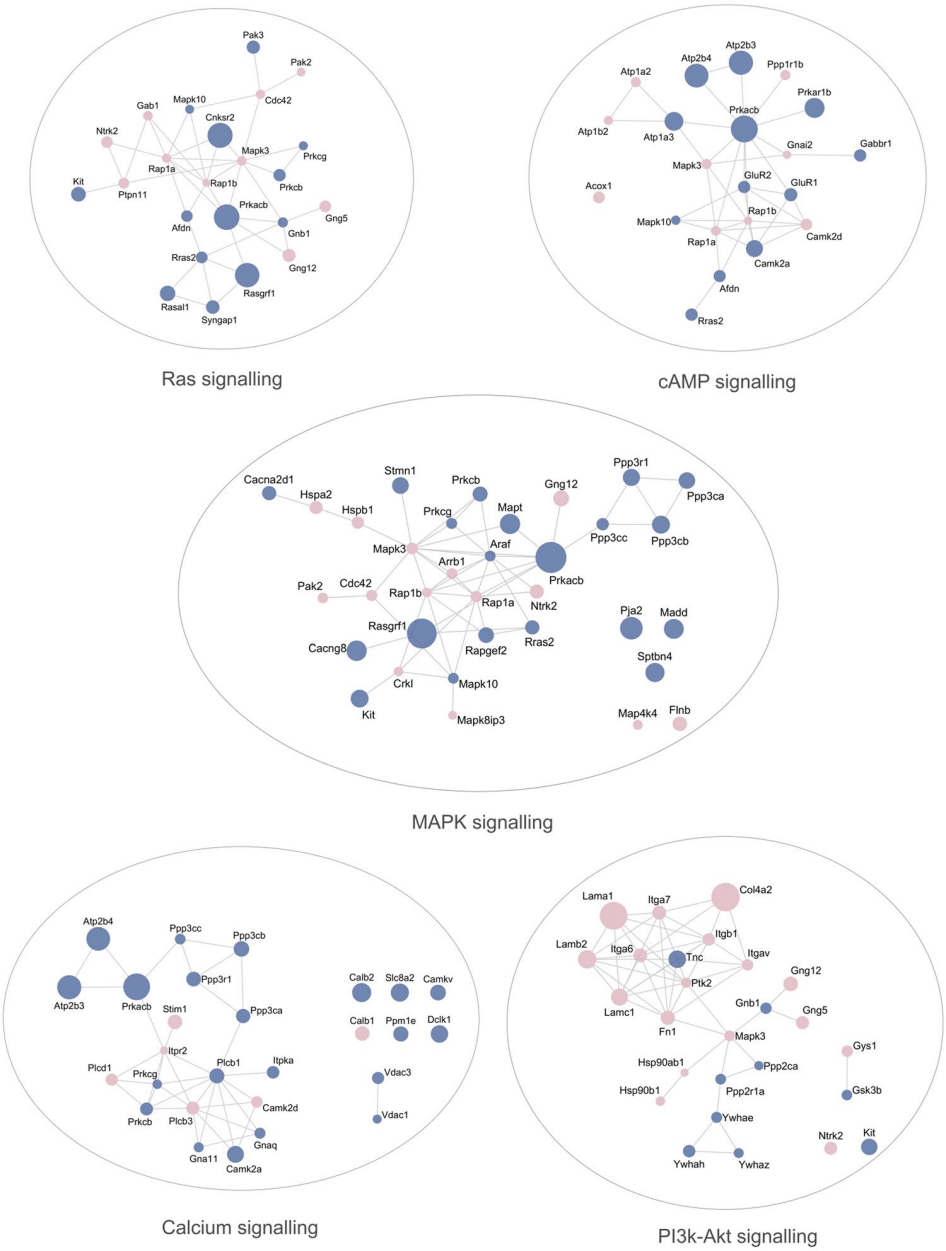
Map of protein–protein interactions of proteins differently abundant between females and males annotated by KEGG within signal transduction pathways. Node colors indicate increased abundant proteins in female (pale pink) and male (blue) hippocampus, and node size indicates different levels of fold change relative to each individual network. STRING online database were used to obtain protein–protein interactions and Cytoscape 3.7.1 for visualizing networks of attributed data. For more details, see Supplementary Dataset 3. Statistical analyses were performed by using Student's *t* test followed by multiple testing corrections using the Benjamini–Hochberg procedure for false discovery rate (n = 5 biological replicates, each containing 3 slices).

Signal transduction controls many aspects of brain function. The specific regulation of synaptic connectivity, axonal growth, cytoskeletal dynamics, neural protection, mitochondrial function, and many other pathways are dependent on a myriad of signal transduction pathways. The differential abundant proteins annotated for each signal transduction pathway showed a wide ranging pattern of response in males and females (Fig. 5, Suppl. Figure 5, Suppl. Dataset 3). The expression of extracellular matrix molecules associated with phosphatidylinositol 3′-kinase (PI3K)-Akt signaling and those that connect the extracellular matrix with the actin cytoskeleton inside the cell were upregulated in females (Suppl. Figure 6).

**Figure 6:**
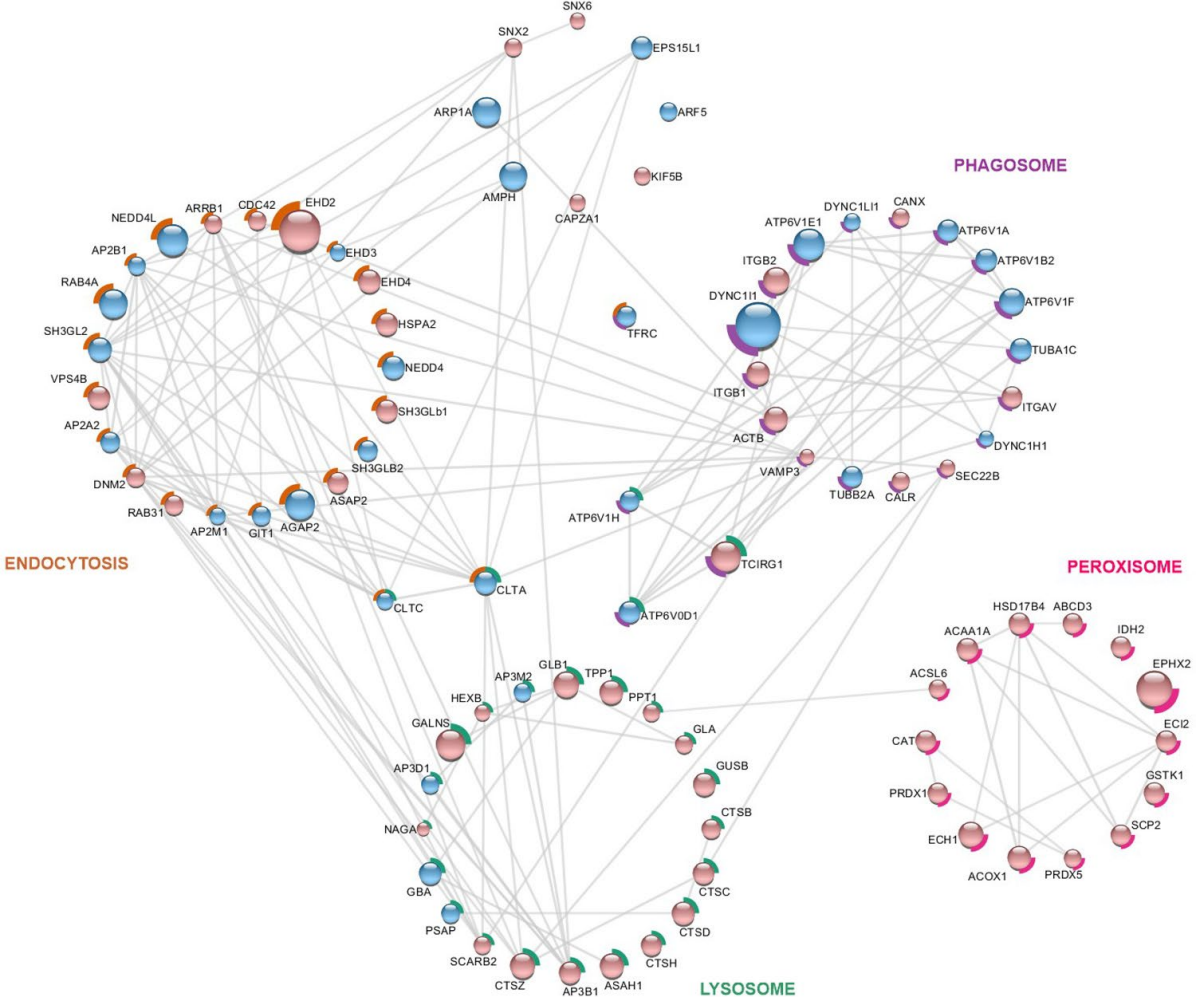
Network analysis of differently abundant proteins annotated to cellular process pathways according KEGG pathway database. Using StringApp, a Cytoscape app, functional enrichment analyzes were retrieved from the whole STRING network of cellular processes entries classified according to BlastKOALA. A subset of networks resulted from the enriched terms *endocytosis* (orange contour), *lysosome* (green contour), *peroxisome* (pink contour) and *phagosome* (purple contour). Node colors indicate increased abundant proteins in female (pale pink) and male (blue) hippocampus and node size indicates different levels of fold change relative to each individual network. For more details, see Supplementary Dataset 2. Statistical analyses were performed by using Student's *t* test followed by multiple testing corrections using the Benjamini–Hochberg procedure for false discovery rate (n = 5 biological replicates, each containing 3 slices).

#### Proteins related to cellular transport and membrane-enclosed organelles

The analysis of cellular transport and membrane-enclosed organelles revealed some patterns (Fig. 6, Suppl. Dataset 2). In endocytosis and phagosomes, females and males present a similar distribution of proteins related to both processes. Components of adaptor protein complex-2 (AP2A2, AP2M1 and AP2B1) and clathrins (CLTC and CLTA) in endocytosis, and tubulin (TUBA1C and TUBB2A) and dyneins (DYNC1L1 and DYNC1H1) in phagosomes were some of the differently abundant proteins upregulated in males. In lysosomes, most of the proteins increased in females, including cathepsins (CTSB, CTSD CTSH and CTSZ) and proteases such as dipeptidyl peptidase 1 (CTSC) and tripeptidyl peptidase (TPP1). Every identified peroxisome-related protein increased in females, including catalase (CAT) and peroxiredoxin 1 and 5 (PRDX1 and PRDX5, respectively) (Fig. 6).

#### Markers for neuronal and glial cell types

**Figure 7:**
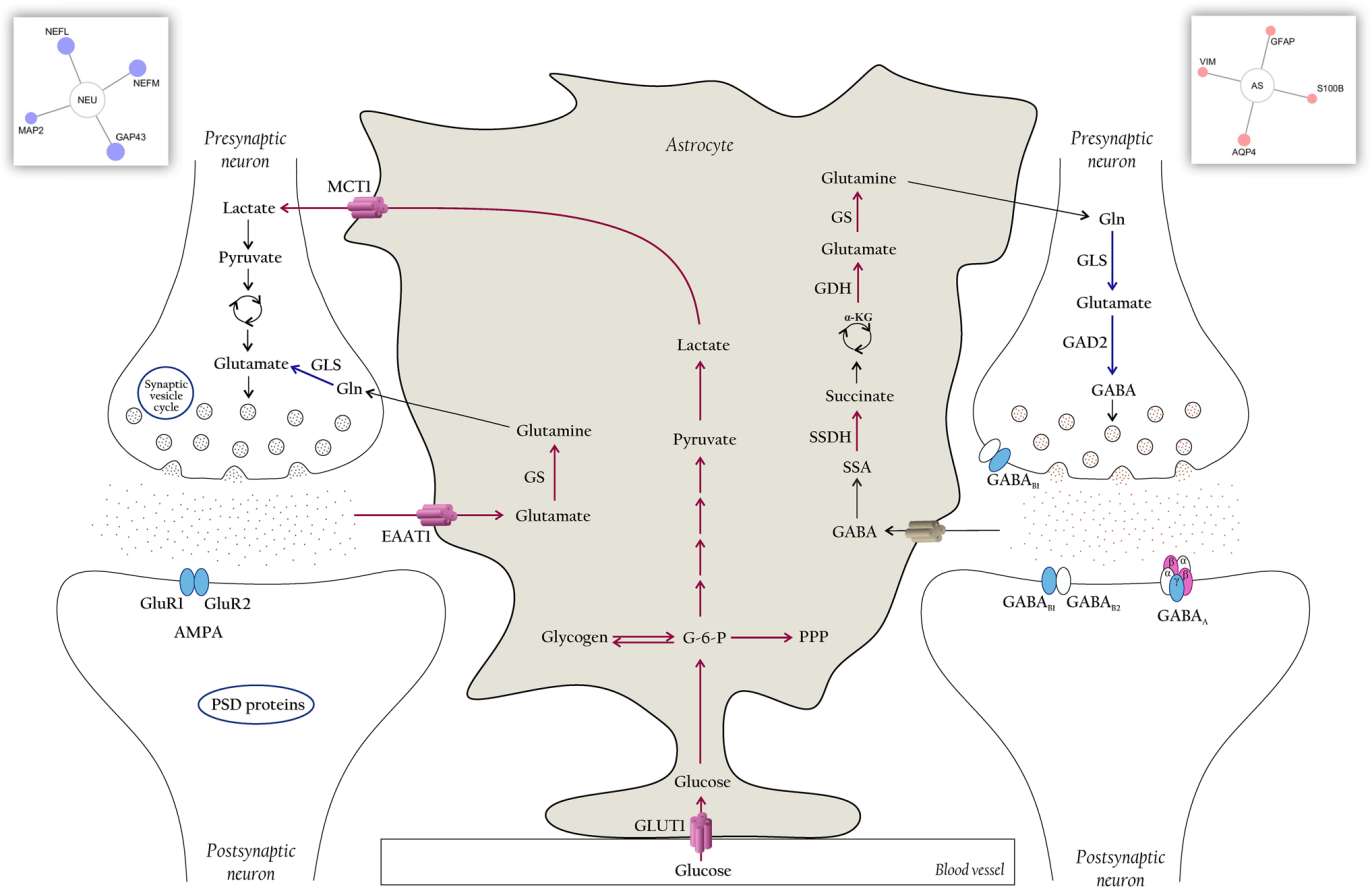
Influence of sex on glutamate-glutamine cycle. Schematic representation of glutamate/GABA-glutamine cycle depicting the metabolic interactions between a glutamatergic neuron, a GABAergic neuron and an astrocyte. Predominant pathways and reactions in females and males are indicated by pink and blue arrows, respectively. Increased abundant transporters and neurotransmitters subunits are evidenced by pink (females) or blue (males) colors. For more details, see Supplementary Information.

It is important to emphasize the sex differences in already established markers of specific cell types from the CNS. The neuronal markers of mature neurons microtubule-associated protein (MAP2, 1.93-FC), neuromodulin (also known as growth-associated protein 43, GAP43, 3.16-FC), and the intermediate filament proteins, neurofilament medium (NF-Medium, 3.04-FC) and light (NF-Light, 3.04-FC) were more abundant in males. On the other hand, the water-specific channel aquaporin-4 (AQP-4, 2.18-FC), S100 calcium-binding protein B (S100b, 1.56-FC) and the intermediate filaments vimentin (VIM, 1.61-FC) and glial fibrillary acidic protein (GFAP, 1.56-FC), well-known astrocytic markers, were upregulated in females (Fig. 7, Suppl. Dataset 3).

### Flow cytometry assay

**Figure 8:**
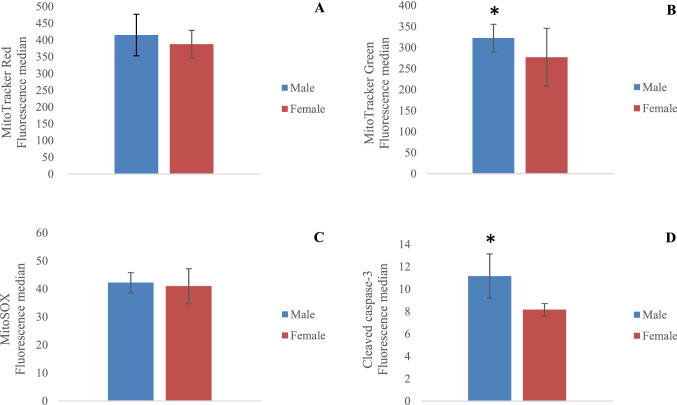
Flow cytometry analysis of mitochondria from female and male organotypic hippocampal slice cultures. Fluorescence median for mitochondrial membrane potential using MitoTracker Red (**A**), mitochondrial mass using MitoTracker Green (**B**), superoxide production using MitoSox (**C**) and cleaved caspase-3 (**D**). Significant differences between females (n = 5 biological replicates, each containing 3 slices) and males (n = 6 biological replicates, each containing 3 slices) were revealed by Student’s *t* test. *denotes *p* < 0.05.

Figure 8 summarizes the results of cells labeled with MTR (Fig. 8A), MTG (Fig. 8B), MitoSOX (Fig. 8C) and caspase-3 (Fig. 8D). No differences between females and males were detected in either MTR (t(9) = 0.841, *p* > 0.05) or in MitoSOX (t(9) = 0.417, *p* > 0.05), indicating no relevant variance in mitochondrial membrane potential or superoxide production. An increase in MTG fluorescence in males (t(9) = 1.359, *p* < 0.05) might represent an increase in mitochondrial population. The higher fluorescence intensity of cleaved caspase-3 (t(9) = 3.557, *p* < 0.05) in males revealed an increment of the key mediator of neuronal programmed cell death. Densitometric quantification of PI intensity revealed no significant cell death in both female and male slices, attesting to the viability of the hippocampal slice culture (*data not shown*). It was crucial to avoid damaged slices entering the following experiments, as cell death may falsify the proteomic and cytometry results.

### TEM images of hippocampal slice culture

**Figure 9:**
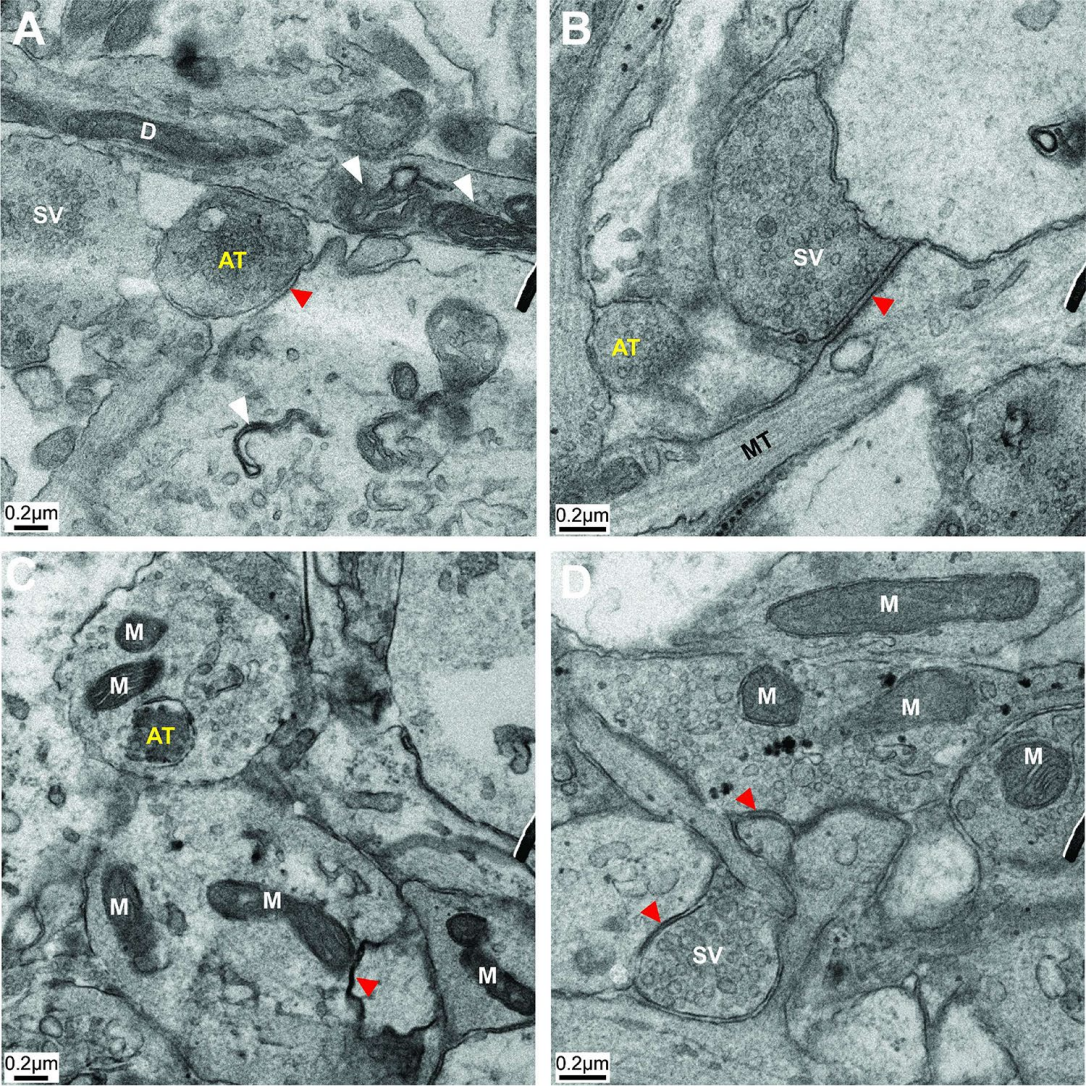
Representative electron micrographs of synapses from male (**A**, **B**) and female (**C**, **D**) hippocampal area CA1. Axon terminal (AT), Dendritic spine (D), Mitochondria (M), Synaptic Vesicle (SV), Microtubules (MT), Myelin sheaths (white arrow), Postsynaptic densities (red arrow). Scale bar: 0.2 µm.

In order to verify any morphological differences at synapses that could contribute to understanding the distinct metabolic pattern between females and males, sections in which synapses at hippocampal area CA1 appeared were simply evaluated. We used the qualitative criteria of the presence of PSD and synaptic vesicles and found that sections obtained from female hippocampal area CA1 present a high density of mitochondria inside synaptic vesicles at the axon terminal, whereas in male synapses, mitochondria were poorly present or even absent (Fig. 9). Although the result is impressive, we cannot claim this is a widespread phenomenon, as this is a series of qualitative observations instead of a quantitative description.

### Data availability

All raw MS data files (.raw files format) used for protein identification and quantification were deposited in the Mass Spectrometry Interactive Virtual Environment (MassIVE, UCSD, California, USA) data repository under the accession code MSV000084210 (http://massive.ucsd.edu/ProteoSAFe/QueryPXD?id=PXD015041). The data that support the findings of this study are available from the corresponding author upon request.

## Discussion

The purpose of this study was to investigate the impact of sex chromosomes on regular hippocampal cellular function of organotypic-cultured slices from female and male rats. Mass spectrometry-based proteomics was employed to obtain a large-scale comparison of the changes in protein abundance, providing the most comprehensive list of differences between the sexes in the hippocampus. A total of 1286 protein entries presented significantly regulated abundance in one of the two conditions (females or males). Most of them were upregulated in females. The culturing of organotypic slices is a method that preserves the contact and synaptic organization of different cell populations and, because of that, neural type-specific inference is a highly delicate issue in the present study. Despite this, it must be highlighted that the large amount of proteomic data obtained from each sex analysis allows us to suggest that there is a greater prevalence of astrocytic metabolism in females and more significant neuronal metabolism in the hippocampus of males.

### Mass spectrometry-based proteomics

The detailed analysis of differentially abundant proteins revealed a consistent upregulation of glucose usage in females and oxidative metabolism in males. It is important to highlight that astrocytic cells primarily metabolize glucose via glycolysis, yielding lactate and glycogen for storage^17,18^, whereas neurons are dependent on oxidative phosphorylation to generate energy.

A preference for glucose flux through the glycolytic, PPP and glycogen pathways was observed in the female hippocampus. The greater abundance of glycolytic enzymes in females was accompanied by pyruvate, preferentially converted to lactate in the cytosol. Both isoenzymes, LDHA, found in lactate-producing cells and predominantly expressed in astrocytes, and LDHB, expressed in lactate-consuming cells such as neurons^19^, were upregulated in females. This result may suggest a continuous synthesis and usage of energy by astrocytes and neurons. The transference of energy in the form of reducing equivalents from cytosol to mitochondria seems to be effectively done by cytosolic glycerol 3-phosphate dehydrogenase (GPD1) in oligodendrocytes and astrocytes^20–22^. The upregulation of GPD1 in females may represent a preferential connection between glycolysis and oxidative phosphorylation, compared to the usual malate-aspartate shuttle.

Energy metabolism in males reflected that described for neuronal demands in the literature, with high abundance of PDH, some of the TCA cycle proteins and oxidative phosphorylation subunits. While there is compartmentation of enzymes and transporters between astrocytes and neurons that determines their specialized metabolism, brain cells present a complementary integrated function. The astrocyte-neuron lactate shuttle (ANLS) hypothesis proposed by Pellerin and Magistretti^17^ states that, during brain stimulation, glutamatergic activity triggers glucose uptake from astrocytes to produce lactate via aerobic glycolysis and subsequent uptake by nearby neurons to serve as major fuel during activation. Such metabolic responses to activation rendered the distinct metabolic phenotypes between neurons (mainly oxidative) and astrocytes (mainly glycolytic)^23^.

At the same time, neurons not only express all glycolytic enzymes but the expression of mRNA in most of them is also greater than astrocytes^24,25^. In line with this, there is strong evidence for glycolysis and respiration upregulation in both neurons and astrocytes in response to brain stimulation^26,27^, with neuronal glycolysis overcoming its oxidation^28^. However, partly counteracting the ANLS model, neurons release lactate in extracellular fluid instead of uptaking it, providing a rapid response to increased energy demand. Interestingly, PFK-1 was the only glycolytic enzyme upregulated in males. The phosphorylation of F6P to F1,6BP by PFK-1 is essentially irreversible and is considered the first point of commitment of glucose to the glycolytic pathway^29^. Three different PFK-1 isoforms are described for rat brain: C (brain), M (muscle) and L (liver) types, which form a tetrameric protein of any combination of the three subunits^30^. Besides the allosteric regulation by several metabolites and post-translational modification, PFK isoforms are differentially distributed in different brain cells, allowing tight control over glycolytic flux^31^. The greater abundance of PFK-1 may probably reflect the important role of the enzyme in the regulation of glucose metabolism in males and the need for a rapid response to the energy status of neurons mainly under brain activation. In situ hybridization experiments, demonstrating that PFK level is higher in neurons than in glial cells, seem to support this idea^31,32^.

The malate-aspartate shuttle is considered the most important shuttle in mature neurons and synaptic terminals^33,34^, and its activity is extremely low or non-existent in astrocytes^35,36^. The continuous transfer of reducing equivalents from the cytosol to mitochondria by the MAS is essential to provide pyruvate to mitochondrial oxidative metabolism, producing the bulk of ATP to sustain synaptic transmission demands^37,38^. The shuttle is composed of both the cytosolic and mitochondrial forms of MDH and GOT, as well as two calcium-binding mitochondrial carriers, for oxoglutarate/malate (OGC/Slc25a11) and for aspartate/glutamate (AGC1/Aralar1/Slc25a12 or AGC2/Citrin/ Slc25a13)^39,40^, all of them upregulated in males. Interestingly, both AGC1 mRNA and protein are highly enriched in neurons, precisely in cytochrome oxidase-rich neurons, probably reflecting the main function of MAS in providing pyruvate and in fueling energy-demanding functions of neurons^35,39,41^. The higher abundance of some subunits of all mitochondrial respiratory chain complexes, along with the phosphate carrier protein and mitochondrial creatine kinase, reinforces the impression of higher neuronal metabolism in males compared to females.

Dependency of neurons on oxidative phosphorylation for energy production relies on the well-known byproduct of oxygen metabolism: the generation of reactive oxygen species (ROS) by the electron transport chain of mitochondria. In this context, the assembly of complex I with complex III into a supercomplex is known to regulate electron transfer efficiency and decrease ROS production^42^. This assembly is dependent on complex I subunit NDUFS1. In neurons, supercomplex-assembled complex I abundance is positively associated with overexpression of NDUFS1, while in astrocytes, the high proportion of free complex I is related to downregulation of NDUFS1. In our data, the 48% increased abundance of NDUFS1 in males may support the perception that males present a predominantly neuronal metabolism. It is known that both high complex III abundance and the amount of sequestered complex I in neurons contribute to their high respiration rate^42^. The results are consistent with the neurons’ dependence on oxidative phosphorylation.

Although the adult mammalian brain is highly dependent on oxidative metabolism of glucose, FAs are readily oxidized by astrocytes and neural progenitor cells^43^ to generate ketone bodies to replace glucose as major source of energy for neurons during energy deprivation^44–46^. In our study, data obtained from the hippocampus of females was consistent with astrocyte FA metabolism. The increased abundance of CPT1a and CPT2 was followed by high levels of β-oxidation enzymatic machinery. In agreement with such idea, a recent work reported that CPT1a and CPT2 are highly enriched in hippocampus and are present specifically in astrocytes and progenitor cells, not neurons, microglia, or oligodendrocytes^43^. Branched- and very long-chain FAs, on the other hand, undergo peroxisomal oxidation to be shortened and delivered to mitochondria for full oxidation to CO_2_ and H_2_O^47^. This seems to be the case for females, as evidenced by increased abundance of the peroxisomal oxidation pathway enzymes, acyl-CoA oxidase (ACOX1) and thiolase (ACAA1), in addition to the ABCD3 transporter, required to import β-oxidation substrates.

Short-chain, medium-chain and polyunsaturated FAs are key components of biological membranes, and have many functions in brain health and homeostasis^48^. Five isoforms of the acyl-CoA synthetases, required for activation of short-chain (ACSS1) and long- to very-long chain FAs (FATP1, ACSBG1, ACSL6), were found upregulated in females. In the brain, ACSL6 is critical to maintaining the high levels of one of the most abundant polyunsaturated FAs in the brain, docosahexaenoic acid (DHA)^49,50^. ACSL6 mRNA and protein abundance are highly enriched in the CNS, and total body germ-line deletion of ACSL6 (ACSL6^−/−^) proved to cause increased microglia activation and astrogliosis^49^. The dynamics of cellular phospholipids turnover seems to be more intense in females than males. Through de novo synthesis (Kennedy pathway), glycerophospholipids are produced from G3P, while at the same time, deacylation-reacylation reactions catalyzed by phospholipases and lysophospholipid acyltransferases remodel glycerophospholipids already incorporated into cellular membranes (Lands cycle)^16,51^. The acyltransferases LPCAT3, LPCAT4 and AGPAT3 upregulated in females are especially important to the production of polyunsaturated fatty acids (PUFAs) containing glycerophospholipids, such as arachidonic acid (AA), linoleic acid and DHA, known to be major sources of bioactive lipids and endocannabinoids^52,53^. Under stimulation, as in inflammation and sepsis, AA can be released from phospholipids by phospholipase A2, making it available for eicosanoid synthesis, which is involved in a plethora of physiological and homeostatic processes^54^. The significant rise in downstream AA oxidation pathways resulting in the production of prostanoids, leukotrienes, hydroxyeicosatetraenoids and epoxyeicosatrienoids (EETs) may evidence the biological importance of eicosanoids in female neural cells. There is robust evidence that astrocytes are responsible for locally regulating blood flow by releasing vasodilator substances such as prostaglandin E2 (PGE_2_), EETs, nitric oxide, or vasoconstrictors such as 20-hydroxyeicosatetraenoic acid (HETE) in response to transient Ca^2+^ changes during synaptic activity^55–59^. Under metabotropic glutamate receptor activation, the lowered oxygen availability and elevated Ca^2+^ concentration in astrocytes promote upregulation of glycolysis, lactate release and PGE_2_ accumulation, facilitating astrocyte-mediated vasodilation^60^. Lipid metabolism data in females appears to be in line with astrocyte metabolism, and its ability to adjust activity-dependent cerebral blood flow, according to metabolic demands elicited by neuronal activity.

Glutamate and GABA are the major neurotransmitters in the mammalian brain, mediating excitatory and inhibitory signaling, respectively. Once released at brain synapses, glutamate is preferentially taken up by glial cells by EAAT1. Then, virtually most glutamate released is converted back to glutamine by the astrocyte-specific enzyme GS and transferred back to excitatory/inhibitory neurons, in a process called the glutamate-glutamine cycle^61,62^. Astrocytes and neurons can also oxidatively metabolize glutamate via the TCA cycle^63,64^ to provide energy during normal metabolism^61,65,66^. Glutamate recycling is tightly coupled to MAS^67^, essential to diverting carbon atoms of glutamate from catabolism in the mitochondrial matrix^68^, maintaining the neuronal neurotransmitter pool of glutamate. When analyzed separately, males and females possess the selective cellular distribution of some proteins associated with the glutamate-glutamine cycle attributed to neurons and astrocytes, respectively, as summarized in Fig. 7.

In GABAergic neurotransmission, the concerted action of GABA-T and SSDH oxidizes GABA, picked up by astrocytes, turning it into succinate. Succinate can either exit the TCA cycle as malate and be decarboxylated to pyruvate by cytosolic malic enzyme (highly enriched in astrocytes) or can be converted to glutamine via α-KG and glutamate^69, 70^. Once formed, glutamine is transported from astrocytes to neurons and converted back to glutamate via deamination by GLS, packaged and stored into vesicles^71^. The synthesis of GABA from glutamate requires an additional step, catalyzed by GAD2, mainly expressed in GABAergic neurons^62,69^. On the other hand, neurons are not able to synthesize glutamate, glutamine and GABA from glucose, since PC, responsible for adding net carbon to the TCA cycle (anaplerosis) and upregulated in females, is only expressed in astrocytes^72^.

The favorable neuronal metabolism observed in males corroborates the highly upregulated pre- and postsynaptic associated proteins detected. Numerous proteins from the presynaptic terminal involved in a process called the synaptic vesicle cycle^73^, and components of the GABA ionotropic and metabotropic receptors at the postsynaptic neuron, were both more abundant in males. The same was true for several proteins associated with postsynaptic density, a macromolecular complex of proteins at the postsynaptic membranes of excitatory synapses, consisting of glutamate receptors, adhesion proteins and channels, scaffolding proteins, kinases and phosphatases responsible for triggering signal transduction cascades^74^. Those results highlight once again the significantly neuronal metabolism in the hippocampus of males.

Another set of proteins required for neuronal circuit formation as guidance receptors that regulate cellular migration, axonal navigation, and synapse formation and maturation were also predominant in males. The question arises of whether the preparation of organotypic slice culture could induce those changes in axonal guidance proteins. Cultured slices undergo some anatomical remodeling after the slicing procedure. The loss or elimination of target innervation due to axotomy leads the damaged axons to recover and reroute their processes to form new neuronal connections^75,76^ by stimulation of neurite sprouting during the first days in culture^13,77^. Microglial cells, activated following explantation, progressively return to a resting state after about a week in vitro^78^. Most dead cells and tissue debris caused by the slicing procedure disappear after 1–2 weeks in vitro^79^. In addition, the slice has time to recover from the altered metabolic state caused by the enzymes and ions released during the first days after explanting^76,80^. That is one of the reasons why slices need to be cultured for at least ten days to stabilize intrinsic axonal projections^13^. In the present study, slices were cultured for 14 days to guarantee structural and synaptic organization of the original tissue in the three-dimensional system.

The metabolic interaction between astrocytes and neurons is present in the detoxification system, particularly glutathione (GSH) metabolism. In this respect, the huge majority of identified enzymes comprising thioredoxins, peroxiredoxins and the glutathione system, vital in maintaining the redox balance in the CNS^81,82^, were increased in females (Fig. 3). Although neurons are particularly sensitive to oxidative stress^83^, they are highly dependent on astrocytes to produce their own GSH content^84^. Astrocytes produce and efficiently export GSH and its precursors to extracellular space, of which the latter are crucial to neuronal GSH synthesis^85^. Therefore, astrocytes normally present GSH levels, total glutathione (GSH and GSSG) and GST activity greater than neurons^85,86^. In addition, astrocytes are those that express a higher PPP-rate limiting step in G6PDH and PPP activity^87,88^, although in neurons the low glycolytic rate of glycolysis could result in the diversion of G6P towards PPP to generate NADPH, and thus to efficiently maintain reduced GSH levels^88,89^. At the same time, enzymes linked to the regulating of peroxide levels within cells, such as peroxyredoxins^90–92^ and catalase, were greater in females, which seems to contribute to the cell defense system against oxidative damage in their hippocampus.

Flow cytometry experiments revealed that levels of cleaved caspase-3, the active form of caspase-3, were upregulated in males. Despite its classic role in cell death, a limited caspase activation in the young adult brain is proven to modulate synaptic plasticity. There is evidence that caspases can perform non-apoptotic functions, especially in learning and memory by influence on long-term potentiation (LTP) and long-term depression (LTD)^93^. During LTD, the cleavage of specific substrates, such as AMPA receptor subunit GluR1, GAP43 and Akt1, results in AMPAR internalization in the postsynaptic membrane, causing synapse weakening and its elimination^94,95^. The transient activation of caspase-3 is associated with moderate activation of the mitochondrial apoptotic pathway with cytochrome *c* release, without causing cell death after the stimulation of short-term glutamate NMDA receptors in CA1 hippocampal neurons^95^. The several upregulated pre- and postsynaptic associated proteins related to glutamate neurotransmission found in males seem to support the signaling of activated caspase during circuit refinement of neurons.

The diversity of synaptic transmission from neuron-to-neuron and from synapse-to-synapse in vertebrate networks is necessary for signal processing that produces the appropriate animal behavior^96,97^. In cultured hippocampal neurons, an identical stimulation can produce varied responses in synaptic strength that are correlated to mitochondrial motility in axons^98^. Upon stimulation, enhancing mitochondria passing through boutons increases the release of synaptic vesicles, mainly by supplying ATP to synaptic transmission; the absence of a stationary mitochondrion within an axonal terminus reduces local ATP supply^98^. Accordingly, increased glucose availability^99^ and changes in cytosolic Ca^2+^^100,101^ cause growth in the number of stationary mitochondria at active synapses, altering ATP homeostasis in axons to match the high energy demand. Although the total number of mitochondria, estimated by mitochondrial mass fluorescence in the hippocampal slices, was more significant in males, a visual inspection of functional synapses on TEM revealed a greater presence of mitochondria in the CA1 of the female hippocampus. It must be highlighted that mitochondrial distribution is regulated by multiple dynamic parameters in response to physiological demands^102^. Here, hippocampal slices were analyzed at resting state, in which distant mitochondria may be sufficient to supply ATP for basal activity, at least in males. The dynamics of mitochondrial distribution in neurons from males and females in response of local electrical stimulation should be done in the future to clarify the possible influence of sex on mitochondrial dynamics from organotypic slices.

The results presented here point to clear sex differences in the proteome of cultured hippocampal slices. The perception that oxidative neuronal metabolism predominates in males, whereas aerobic glycolysis takes place in female cells, was followed by many other findings in pathways known to be cell-specific. One could ask if the sex differences in protein abundance detected in the present study could be due to differential densities of neuronal and non-neuronal cell types. This claim may be partly true. The number of glial cells and neurons varies across the brain and according to different strata at Ammon’s horn (CA) and dentate gyrus within the hippocampus from a mouse brain^103,104^. The overall astrocyte to neuron ratio in the hippocampus is 0.68 in male adult animals^103^, whereas young female C57Bl6/ J mice have on average 20% more microglia and astrocytes in the CA1 and dentate gyrus regions than age-matched males, a difference also observed as aging progresses^105^. Even so, the fold-change of protein abundance observed in many pathways ranged from approximately 1.10–7.0 times between males and females. Given that, we assume that sex-specific differences in the hippocampus proteome are quite likely to be associated with greater neuronal metabolism in males and higher astrocytic metabolism in females, than with a difference in the ratio of neural cells.

Cell cultures are indispensable tools for basic research, providing substantial information on cell biology, drug screening, toxin testing, or other cell-based assays. However, cells in culture are exposed to conditions that add significant variables, such as artificial medium formulations that do not recapitulate the in vivo conditions. Most studies culture brain slices in 25% horse serum. Serum provides proteins, carbohydrates, lipids, minerals, growth factors and hormones. Despite the serum-based media may contain undefined animal-derived products and might not be adequate to explore sex differences, the purpose of our study was to maintain the same environment for hippocampal slices used in experimental protocols that reproduce neurological diseases largely used in the literature. With increasing rational use of male and female individuals to study neurodegenerative diseases with sex-biased incidence, exploring sex differences per se is essential to understand the results obtained in those experiments and for future comparisons between sexes in different models of brain injury. Nevertheless, future experiments should be performed to compare different types of culture media to define the best formulation to study sex differences in experimental models of neurological diseases.

In conclusion, we have shown an extensive characterization and interpretation of sex-specific differences in the proteome from cultured organotypic hippocampal slices that will provide a broad basis for future analyses. The lack of female data represents an obstacle to the comprehension of sex influence on several neurological diseases. Understanding those mechanisms is important both to better understand the dynamics of physiology and pathophysiology of the nervous system and to further define new therapeutic strategies delineated for a number of neurological diseases that present a sex-biased incidence. Emerging technologies using recombinant DNA combined with in vivo models may represent an advance in translational research relevant to neurodegenerative disorders.

## Methods

### Animals

Wistar rats were obtained from the Central Animal House of the Biochemistry Department, Institute of Basic Health Sciences, at the Federal University of Rio Grande do Sul. Animals were maintained in a 12/12 h light/dark cycle in an air-conditioned room at constant temperature (22 ± 1 °C), with free access to food (SUPRA, Porto Alegre, RS, Brazil) and water. All procedures were in accordance with the Principles of Laboratory Animal Care (NIH publication No. 85-23) and with the Federation of Brazilian Societies for Experimental Biology. The study was approved by the Animal Research Ethics Committee of the Federal University of Rio Grande do Sul (Protocol. nr. 35489). All efforts were made to minimize animal suffering as well as to reduce the number of animals.

### Preparation of organotypic hippocampal culture

Male and female Wistar rats from 6 to 8 days old were used^106^. The sex of the rat pups was determined based on the anogenital distance, shorter in females than in males. After decapitation, the brain was rapidly removed from the skull and hippocampi from both hemispheres were carefully dissected. Under aseptic conditions, hippocampus sections, 400 µm in thickness, were obtained using a Mcllwain tissue chopper (Mickle Laboratory Engineering Co., Guildford, UK) and collected in an ice-cold dissection medium consisting of Hanks’ Balanced Salt Solution (HBSS) with 25 mM HEPES and 6% glucose. The organotypic slices were carefully placed onto the top of 0.4 µm pore size semipermeable membrane inserts (Milicell, Millipore, USA) previously prepared in six-well plates with 1 mL of warm culture medium below the membrane. Organotypic cultures were maintained in a humidified incubator gasified with a 5% CO_2_ atmosphere at 37 °C. The culture medium consisted of 50% minimum essential medium (MEM), 25% HBSS, 25% heat-inactivated horse serum, supplemented with 36 mM glucose, 25 mM HEPES, NaHCO_3_, 1% Fungizone and 1% Penicillin–Streptomycin, pH 7.3. The media were replaced by fresh solution two times per week for 14 days and all procedures were performed aseptically in a horizontal flow hood.

### Quantification of cell death

The viability of the organotypic culture was assessed by measuring the permeability of the plasma membrane to the normally impermeable fluorescent, DNA-binding dye, propidium iodide (PI—Sigma-Aldrich, USA)^107^. On the 14th day in vitro (DIV), 5 µM of PI were added to the culture medium and incubated for 2 h at 37 °C with 5% CO_2_ in a humidified incubator. Cultures were analyzed under an inverted fluorescence microscope (Eclipse TE 300, Nikon, Tokyo, Japan) using a standard rhodamine filter set. Images were captured using a CCD camera (Visitron Systems, Puchheim, Germany) and analyzed using Scion Image software (Maryland, USA). Quantification of the mean intensity of the PI fluorescence was determined densitometrically, considering the total area of hippocampal tissue.

### Sample preparation for mass spectrometric analysis

After obtaining the fluorescent images, slices were washed with cold PBS to remove the PI. Samples were prepared according to the filter-aided sample preparation (FASP) protocol^108^. Briefly, three fresh slices from each animal in each sex (n = 5 biological replicates, each group) were lysed in a buffer consisting of 0.02 M triethylammonium bicarbonate (TEAB), 0.1 M dithiothreitol (DTT) and 4% sodium dodecyl sulfate (SDS), supplemented with protease and phosphatase inhibitor cocktails, cOmplete and PhosStop, respectively (Roche Diagnostics, Germany)^109^. The lysates were incubated at 80 °C for 10 min and sonicated in an ultrasonic processor (Markson, Model GE 50 T 20 kHz). The resultant homogenates were centrifuged at 18.000 × *g* for 20 min at 4 °C, and protein content of supernatants was determined using the fluorescence spectrometer Qubit (Thermo Fisher Scientific, Massachusetts, USA). Aliquots of 200 µg of protein were subjected to digestion with trypsin (1:80) for 20 h at 37 ºC via FASP^108^. Digested peptides were desalted on C18-reverse phase micro-columns using self-prepared StageTips^110^, resuspended in 0.1% formic acid and quantified by Qubit protein assay for subsequent label-free analysis by mass spectrometry.

### LC–MS/MS analysis

Mass spectrometry (MS) analyses were performed on an Orbitrap Elite (Thermo Fisher Scientific, Massachusetts, USA) coupled on-line to a nanoflow Dionex Ultimate 3000 RSLCnano UPLC system. The chromatography system was equipped with pre-column of 2 cm with an internal diameter of 100 μm (ReprosilPur C18, 5 μm, 120 Å, Dr. Maich GmbH, Ammerbuch, Germany) and analytical column of 35 cm and internal diameter of 75 μm (ReprosilPur C18, 3 μm, 120 Å, Dr. Maich GmbH, Ammerbuch, Germany), both in-house packed. Briefly, 1 µg of peptides in each technical duplicate was automatically loaded into the trap column at a flow rate of 3 µL min^−1^ in 98% buffer A (0.1% formic acid in water) and 2% buffer B (0.1% formic acid in acetonitrile). Eluted peptides were separated at 250 nL min^−1^ flow rate on the analytical column by 5–45% gradient of buffer B for 170 min, followed by a 45–85% gradient of buffer B over 10 min, sustained for 10 min, and then to 2% buffer B over 5 min, followed by column re-equilibration for another 18 min. Acquisition in the mass spectrometer was performed in data dependent acquisition (DDA) mode. The DDA cycle consisted of a full scan in FTMS comprising a 300–1800 m*/z* range under a resolution of 120,000 full width at half-maximum at *m/z* 400. The 20 most abundant ions with the intensity of at least 1000 counts were selected and fragmented by collision-induced dissociation (CID). The fragmentation was performed with a collision energy of 35%, automatic gain control (AGC) of 1 × 10^6^ and acquired in the ion trap analyzer with a 2 m*/z* isolation width and AGC 1 × 10^4^. Dynamic exclusion was set to 90 s. Ions with charge-state of + 1 or undetermined were excluded^109^.

### Database search and quantification

Data were analyzed using Progenesis QI proteomics software (version 1.0; Non-Linear Dynamics, a Waters Company, Newcastle upon Tyne, UK). For peptide identification, MS/MS spectra with *p* < 0.05 (ANOVA) were exported as Mascot generic files (.mgf) and searched with PEAKS Studio 7.0 (Bioinformatics Solutions Inc., Ontario, Canada) using the Uniprot Rat protein database (containing 35,144 sequences downloaded on 03/17/2016). The parameters used were: 10 ppm peptide mass tolerance, 0.6 Da fragment mass tolerance and two missed cleavages allowed. Methionine oxidation and acetylation of protein N-terminal were included as variable modifications, and carbamidomethylation of cysteine was specified as a fixed modification. To assess the false-positive identification rate, the databases used were reversed on-the-fly during the database queries and appended to the original database. Protein identification false discovery rate (FDR) was set to 0.1%. Identifications were re-imported into Progenesis QI for protein quantification. A minimum of two unique peptides were considered for further analyses. Conflicting peptides were automatically detected and manually validated according to the protein score, peptide score and abundance profile. Peptides still in conflict after manual validation were excluded from protein quantification. PCA analysis was performed at both peptide and protein level to enhance pattern recognition and data summary, as well as to detect the eventual presence of outliers^109^.

### Pathway analysis

KOBAS 3.0 Bioinformatics tool (http://kobas.cbi.pku.edu.cn/home.do) was applied to differentially abundant proteins in female and male groups to identify overrepresented processes and functional protein set enrichment in the proteome of each group^112^. The annotated output file of total hippocampal proteins previously identified was used as background. The hypergeometric test / Fisher exact test was used for the enrichment analysis, and the method of Benjamini and Hochberg^111^ was used for correction of false discovery rates (FDR) with a small-term cutoff of 5. Moreover, differentially abundant hippocampal proteins were classified according to their biochemical pathways using KEGG analysis (http://www.genome.jp/kegg/pathway.html) using the freely available BlastKOALA (http://www.kegg.jp/blastkoala/) sequence similarity tool. Proteins annotated in each biochemical pathway were subsequently analyzed on STRING online database (https://string-db.org/) to obtain a high confidence protein–protein interaction (confidence score threshold was set to 0.7).

### Transmission electron microscopy

Hippocampal slices were fixed in 3% glutaraldehyde in 0.1 M sodium cacodylate buffer at pH 7.2, on an ice bath under microwave irradiation^112^, and then rinsed in buffer. Immersed in a fixative, each slice was trimmed manually under a dissecting microscope to leave a block containing especially the CA1 and dentate gyrus areas. Slices were washed in a buffer and post fixed in 1% osmium tetroxide with 1.6% potassium ferricyanide. Conventional dehydration was conducted with ethanol series (25, 50, 70, 80, 95 and 100%). Samples were progressively infiltrated with an Epon-Araldite resin and ethanol in increasing gradients of resin at room temperature and finally embedded in resin, remaining at 60 °C for curing. Subsequently, 1 µm thick sections were obtained from blocks using a Reichert UltraCut S Ultramicrotome and stained with toluene blue for light microscopic examination. Further, 70 nm ultrathin sections were cut and put on coated 50 mesh copper grids (PELCO Grids, Ted Pella). These grids were post stained with uranyl acetate and lead citrate and checked in a Tecnai T12 transmission electron microscope 120 kV (Thermo Fischer Scientific/FEI, Hillsboro, OR). Images were recorded using a Gatan CCD with Digital Micrograph software (Gatan Inc., Pleasanton, CA). Serial EM software (bio3d.colorado.edu/SerialEM) was used to collect wide-field montages for overview TEM images of hippocampal cross-sections. Adobe Illustrator was used for further image processing.

### Flow cytometry

Mitochondrial membrane potential and mass were measured using MitoTracker Red (MTR) and MitoTracker Green (MTG) dyes (Invitrogen, Molecular Probes, Oregon, USA). The content of mitochondrial superoxide was detected using the probe MitoSOX Red (Invitrogen, Molecular Probes, Eugene, OR—USA). To detect apoptosis, cells were labeled with cleaved caspase-3 antibody (Cell Signaling Technology, Massachusetts, USA). Hippocampal slices (approximately 100 mg) were mechanically dissociated in phosphate-buffered saline (PBS) containing 0.1% collagenase type IV, 0.1% DNAse and 0.2% trypsin to favor digestion and the contents were filtered using a 40 µm nylon cell strainer (Cell Filter Strainer—BD Biosciences, California, USA). Each sample (100 µL) was incubated at 37 °C in the dark, with 1 µM MitoSOX for 20 min and 100 nM MTG and MTR for 45 min^11,113^. For apoptosis analysis, cells were centrifuged at 400 × *g* for 5 min, resuspended in PBS containing 0.1% Triton X-100 and incubated with mouse anti-cleaved caspase 3 antibody (1:100), for 30 min at room temperature. Samples were incubated for 30 min with secondary antibody anti-mouse Alexa Fluor 488 (1:100, Invitrogen)^114^. Fluorescence intensities were analyzed using a FACSCalibur flow cytometer (BD Biosciences, California, USA). Cells were gated based on FSC and SSC pattern of the sample cells and 30,000 events were acquired per sample. Controls stained with a single dye were also employed to allow the setting of compensation. Acquisitions were performed using CELLQuest Pro data acquisition (BD Biosciences, California, USA) and analysis by the FCS Express 5 software (De Novo Software, Los Angeles, CA, USA).

### Statistical analysis

Data are represented as mean ± standard deviation (SD), and statistical analyses were performed using the Statistical Package for the Social Sciences software version 23.0 (SPSS Inc. Chicago, IL, USA). Student’s *t*-test was applied to determine statistical differences between experimental groups for cell viability quantification and flow cytometry assays. Data were considered statistically significant when *p* < 0.05. Protein abundance was analyzed by Student's *t*-test using the R programming environment (R Core Team, 2019—http://www.r-project.org), followed by multiple testing corrections using the Benjamini–Hochberg procedure for false discovery rate. A *q* < 0.05 was considered statistically significant.

## Supplementary Information

Supplementary Dataset 1.

Supplementary Dataset 2.

Supplementary Dataset 3.

Supplementary Dataset 4.

Supplementary Figure 1.

Supplementary Figure 2.

Supplementary Figure 3.

Supplementary Figure 4.

Supplementary Figure 5.

Supplementary Figure 6.
