# Supplementary material for: In-depth quantitative proteomic characterization of organotypic hippocampal slice culture reveals sex-specific differences in biochemical pathways
Source: Sci Rep. 2021 Jan 28;11:2560. doi: 10.1038/s41598-021-82016-7 (PMC7844295; doi:10.1038/s41598-021-82016-7)
Supplement: Supplementary file 6 — Supplementary Figure 2. [file 41598_2021_82016_MOESM6_ESM.pdf]

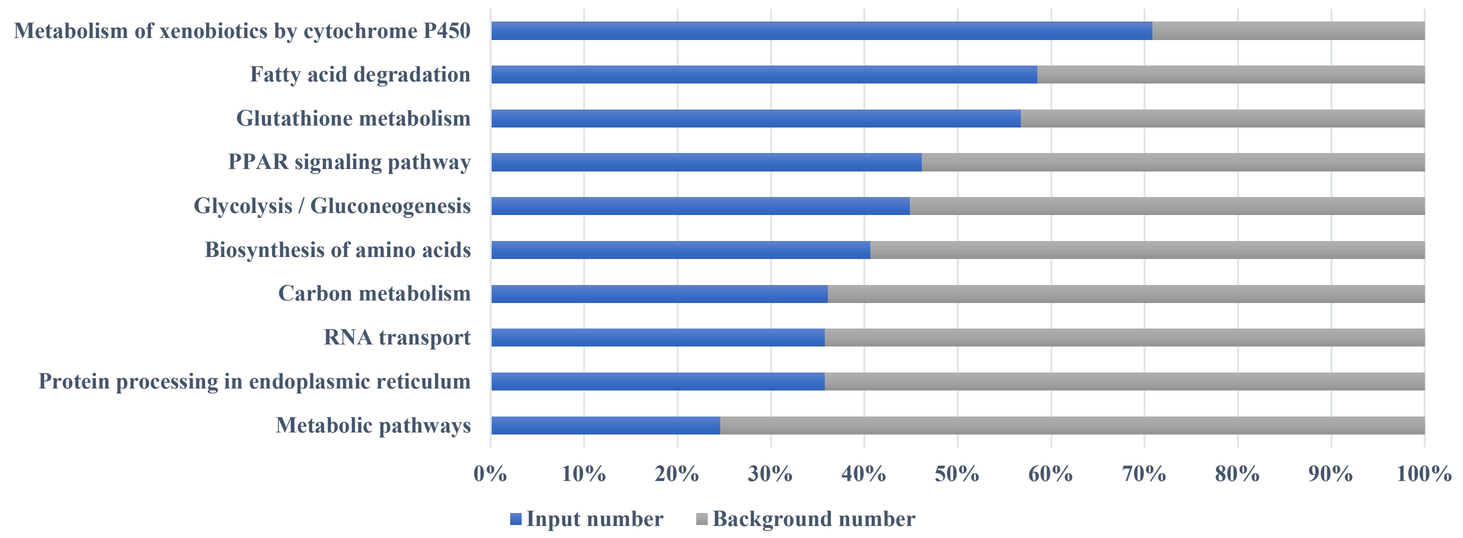

a

In-depth quantitative proteomic characterization of organotypic hippocampal slice culture reveals sex-specific differences in biochemical pathways

S.N. Weis[a], J.M.F. Souza[a], J.B. Hoppe[b], M. Firmino[a], M. Auer[c], N.N. Ataii[c], L.A. da Silva[d], M.M. Gaelzer[e], C.P. Klein[b], A.R. Mól[a], C.M.R de Lima[a], D.O. Souza[b], C.G. Salbego[b], C.A.O. Ricart[a], W. Fontes[a], M.V. de Sousa[a]*
[a]Laboratory of Protein Chemistry and Biochemistry, Department of Cell Biology, Institute of Biology, University of Brasilia, Brazil; [b]Department of Biochemistry, Federal University of Rio Grande do Sul, Brazil; [c]Molecular Biophysics and Integrated Bioimaging Division, Lawrence Berkeley National Laboratory, CA, USA; [d]Laboratory of Electron Microscopy, Department of Cell Biology, Institute of Biological Sciences, University of Brasilia, Brazil; [e]University of Miami, Miller School of Medicine, Miami, FL.

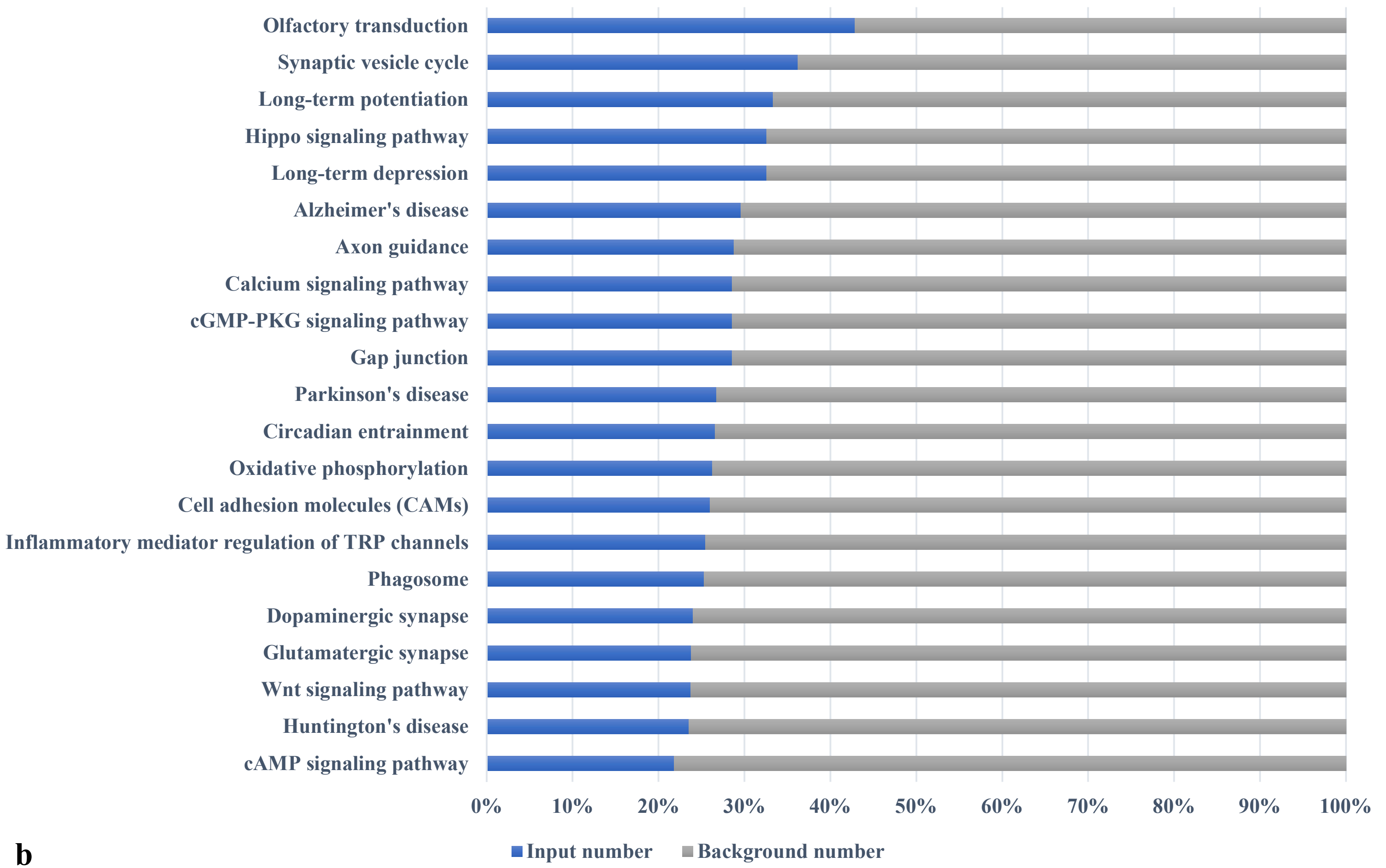
Olfactory transduction
Synaptic vesicle cycle
Long-term potentiation
Hippo signaling pathway
Long-term depression
Alzheimer's disease
Axon guidance
Calcium signaling pathway
cGMP-PKG signaling pathway
Gap junction
Parkinson's disease
Circadian entrainment
Oxidative phosphorylation
Cell adhesion molecules (CAMs)
Inflammatory mediator regulation of TRP channels
Phagosome
Dopaminergic synapse
Glutamatergic synapse
Wnt signaling pathway
Huntington's disease
cAMP signaling pathway
0%
10%
20%
30%
40%
50%
60%
70%
80%
90%
100%
Input number
Background number

b
