# Supplementary figures and images for: In-depth quantitative proteomic characterization of organotypic hippocampal slice culture reveals sex-specific differences in biochemical pathways
Source: Sci Rep. 2021 Jan 28;11:2560. doi: 10.1038/s41598-021-82016-7 (PMC7844295; doi:10.1038/s41598-021-82016-7)

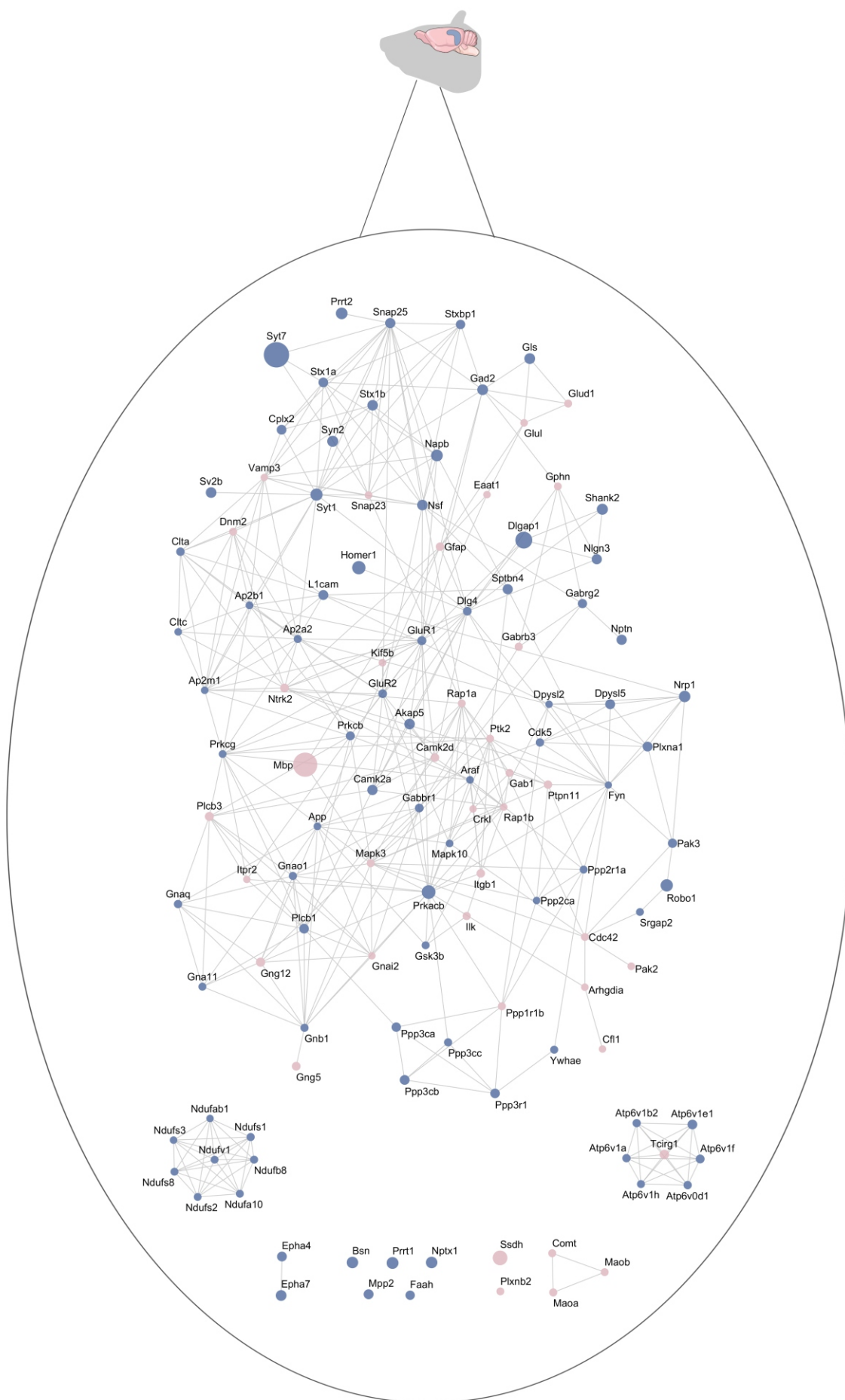

Supplement: Supplementary file 7 — Supplementary Figure 3. [file 41598_2021_82016_MOESM7_ESM.pdf]

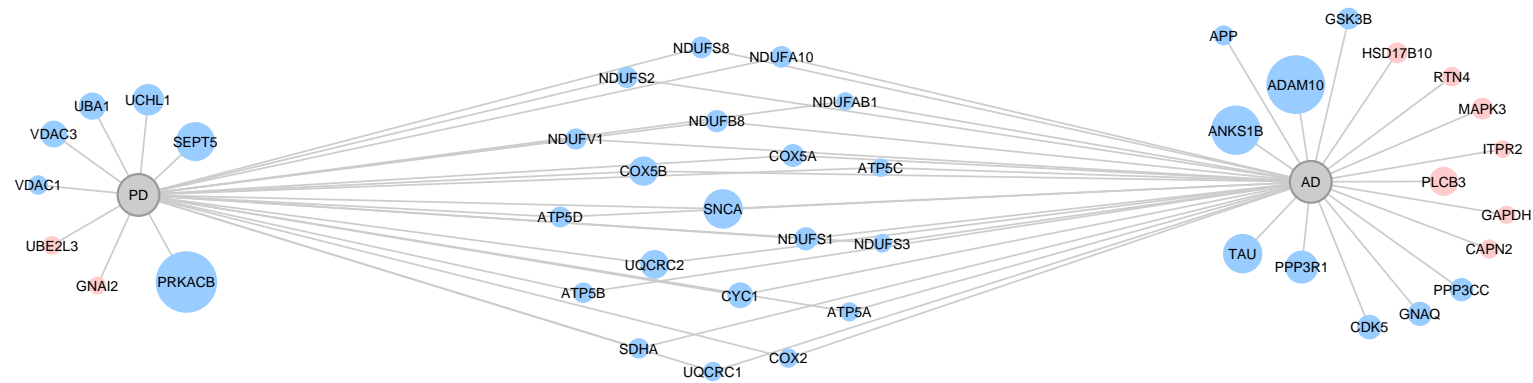

Supplement: Supplementary file 8 — Supplementary Figure 4. [file 41598_2021_82016_MOESM8_ESM.pdf]
