# Supplementary material for: In-depth quantitative proteomic characterization of organotypic hippocampal slice culture reveals sex-specific differences in biochemical pathways
Source: Sci Rep. 2021 Jan 28;11:2560. doi: 10.1038/s41598-021-82016-7 (PMC7844295; doi:10.1038/s41598-021-82016-7)

In-depth quantitative proteomic characterization of organotypic hippocampal slice culture reveals sex-specific differences in biochemical pathways

S.N. Weis[a], J.M.F. Souza[a], J.B. Hoppe[b], M. Firmino[a], M. Auer[c], N.N. Ataii[c], L.A. da Silva[d], M.M. Gaelzer[e], C.P. Klein[b], A.R. Mól[a], C.M.R de Lima[a], D.O. Souza[b], C.G. Salbego[b], C.A.O. Ricart[a], W. Fontes[a], M.V. de Sousa[a]*
[a]Laboratory of Protein Chemistry and Biochemistry, Department of Cell Biology, Institute of Biology, University of Brasilia, Brazil; [b]Department of Biochemistry, Federal University of Rio Grande do Sul, Brazil;
[c]Molecular Biophysics and Integrated Bioimaging Division, Lawrence Berkeley National Laboratory, CA, USA; [d]Laboratory of Electron Microscopy, Department of Cell Biology, Institute of Biological Sciences,
University of Brasilia, Brazil; [e]University of Miami, Miller School of Medicine, Miami, FL.

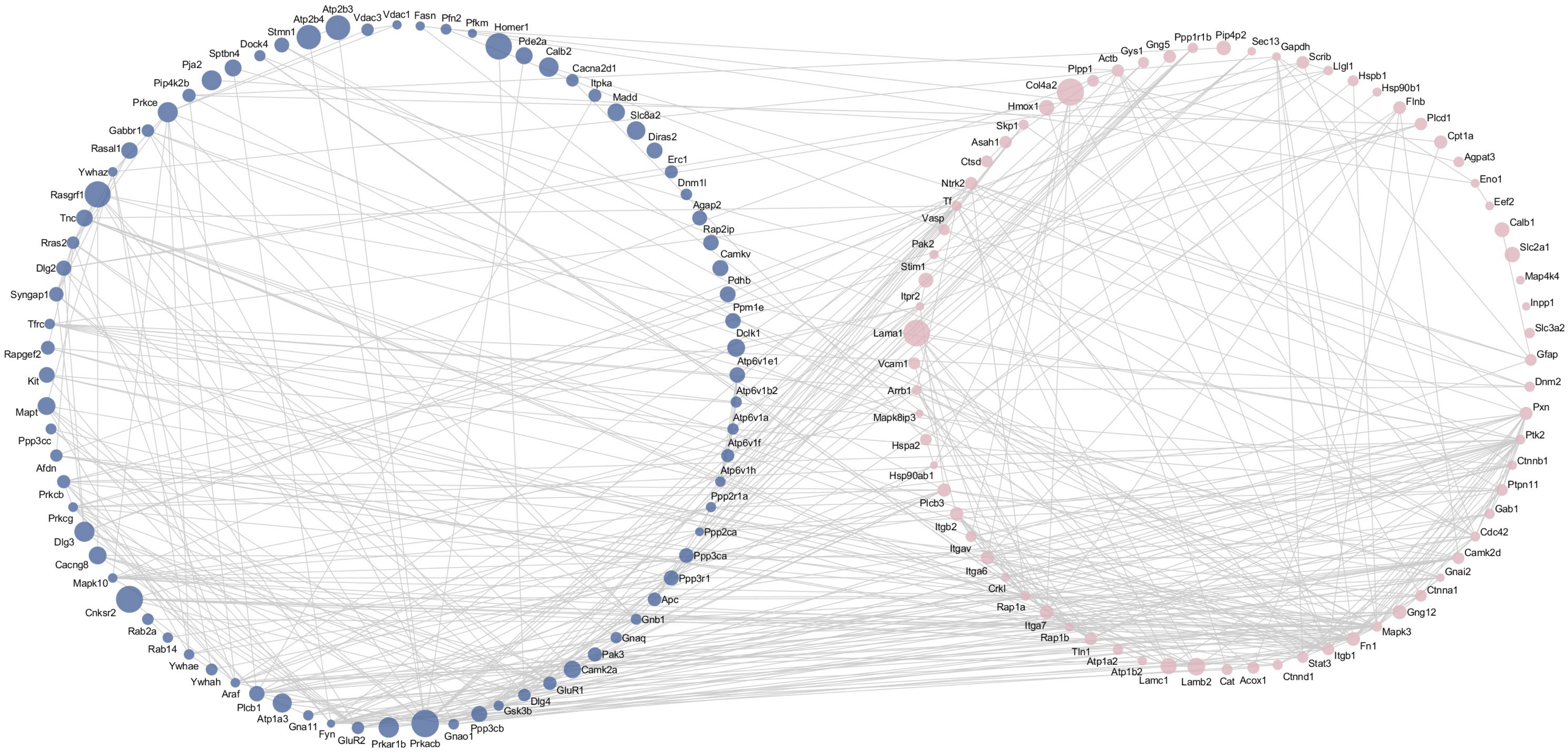

Supplement: Supplementary file 9 — Supplementary Figure 5. [file 41598_2021_82016_MOESM9_ESM.pdf]
